# Long-term outcomes of one-stop unilateral thoracoscopic hybrid ablation for persistent atrial fibrillation after failed catheter ablation

**DOI:** 10.1186/s13019-026-04441-x

**Published:** 2026-06-25

**Authors:** Lina Wang, Xingpeng Liu, Ju Mei, Hao Liu, Changlin Lu, Fengnan Wang, Bingrui Wang, Jingshu Lei, Chen Tan, CHONGYOU LEE

**Affiliations:** 1 Department of Cardiology, Center for Cardiovascular Translational Research, BeijinKey Laboratory of Innovative Drug and Device Translation in Endocrine and Metabolic Diseases, Peking University People’s Hospital, Beijing, 100044 China; 2 Heart Center, HTRM Cardiovascular Hospital, Xuanhui Road, Decheng District, Dezhou City, 253000 Shandong Province China; 3 Department of cardiothoracic Surgery, Xinghua Hospital, School of Medicine, Shanghai Jiaotong University, 200092 Shanghai, China; 4 Department of Cardiology, Beijing Chaoyang Hospital, Beijing, 100016 China; 5 Department of Cardiology, Hebei Yanda Hospital, Langfang, Hebei 065201 China

**Keywords:** Atrial fibrillation, Hybrid ablation, Thoracoscopy, Left atrial appendage, Long-term outcomes

## Abstract

**Background:**

Hybrid ablation is a treatment option for persistent (PsAF) or long-standing persistent atrial fibrillation (LS-PsAF) after failed catheter ablation (CA), but long-term outcomes (> 5 years) and predictors of recurrence after hybrid ablation in this population have not been well characterized in prior studies.

**Methods:**

We retrospectively analyzed 44 consecutive patients with symptomatic PsAF or LS-PsAF who underwent one-stop unilateral thoracoscopic hybrid ablation with left atrial appendage (LAA) management between May 2015 and April 2021. The procedure consisted of same-day epicardial and endocardial ablation. Primary endpoint was defined as recurrence of atrial arrhythmias ≥ 30 s after the 3-month blanking period, regardless of antiarrhythmic drug (AAD) use. Given the limited sample size and number of events, multivariable Cox regression was performed as an exploratory analysis.

**Results:**

Patients (77.3% male; mean age 59.6 ± 8.3 years) had median AF duration of 8.0 years; 97.7% had prior failed CA (mean 1.4 ± 0.7 procedures). At discharge, 93.2% were in sinus rhythm, with no in-hospital mortality or stroke. Over median 5.2-year follow-up, freedom from arrhythmias at 1, 3, and 5 years was 68.2%, 52.3%, and 50.0%, respectively. Recurrence occurred in 22 patients (50.0%), with 63.6% of recurrences within the first year. On exploratory multivariable Cox regression analysis, left atrial (LA) diameter (HR 1.073, 95% CI: 1.001–1.151, *P* = 0.047) showed a potential association with recurrence. LAA pathology revealed myocardial hypertrophy (84.1%), inflammation (65.9%), and occult thrombus was identified in 10.0% of LAA specimens (4/40). No thromboembolic events occurred during follow-up.

**Conclusions:**

One-stop hybrid ablation with routine LAA management was associated with acceptable safety and rhythm control in half of patients with PsAF/LS-PsAF at 5 years within this exploratory cohort. These long-term data suggest hybrid ablation is a viable strategy in this challenging population. Although routine LAA resection/clipping yielded promising histological findings, definitive clinical benefits could not be confirmed within this single-arm cohort. Given the exploratory nature of the analysis and limited sample size, these findings require validation in larger prospective multicenter studies.

**Supplementary Information:**

The online version contains supplementary material available at https://doi.org/10.1186/s13019-026-04441-x.

## Introduction

Atrial fibrillation (AF) affects nearly 33 million people worldwide, with a lifetime risk of 25% after age 40 [1]. Catheter ablation (CA) with pulmonary vein isolation (PVI) is highly effective for paroxysmal AF, but outcomes in persistent AF (PsAF) and long-standing persistent AF (LS-PsAF) remain suboptimal (40%-60% long-term success) [2–4]. This is attributed to a more complex substrate—including atrial fibrosis and extra-PV triggers—that is not addressed by PVI alone [5, 6].

Surgical ablation for AF has evolved considerably since the original Cox-maze procedure. The development of bipolar radiofrequency clamps enabled the less invasive Cox-maze IV, and more recently, standalone thoracoscopic epicardial ablation has emerged as a minimally invasive alternative [7, 8]. However, thoracoscopic epicardial ablation alone has inherent limitations: it cannot reliably achieve bidirectional block across all intended lines, and critical endocardial structures such as the cavotricuspid and mitral isthmuses are inaccessible from the epicardium [8].

Hybrid approaches combining thoracoscopic epicardial and endocardial CA have been proposed to address these limitations, though their optimal timing, lesion set, and long-term efficacy continue to be refined [9–12]. Reported success rates vary widely (27%-94%), reflecting differences in patient selection, lesion sets, and success definitions. Hybrid ablation is also associated with higher complication rates and longer hospital stays than CA alone [12]. However, long-term outcome data beyond 5 years remain scarce. Predictors of late recurrence are poorly defined, and the role of hybrid ablation in patients with prior failed CA—a large real-world population—remains uncertain [9–12]. Therefore, we aimed to evaluate the long-term outcomes of a one-stop hybrid ablation procedure in a real-world cohort of PsAF/LS-PsAF patients with prior failed CA, focusing on arrhythmia-free survival, recurrence patterns, predictors of failure, and procedural safety over > 5 years of follow-up.

## Methods

### Study population

We conducted a retrospective review of 44 consecutive patients with symptomatic PsAF or LS-PsAF who underwent a one-stop hybrid procedure at Hebei Yanda Hospital between May 2015 and April 2021. This single-center study followed an observational design.

PsAF was defined as AF continuously sustained beyond 7 days, including episodes terminated by cardioversion (pharmacologic or electrical) after at least 7 days. LS-PsAF was defined as continuous AF of more than 12 months duration at the time a rhythm control strategy was being considered, consistent with current guidelines.

Inclusion criteria were as follows: (1) age < 75 years; (2) symptomatic PsAF or LS-PsAF; AND (3) meeting one of the following based on AF duration: (a) AF duration < 1 year with at least one prior failed CA; OR (b) AF duration 1–3 years with left atrial (LA) anteroposterior diameter > 50 mm on transthoracic echocardiography; OR (c) AF duration > 3 years (no additional requirements).

These stepwise criteria were intentionally designed to capture difficult-to-treat patients across different dimensions of disease severity: refractory disease (short duration + prior failed CA), advanced structural remodeling (intermediate duration + LA > 50 mm), or long-standing AF (> 3 years). Accordingly, the heterogeneity of our cohort reflects real-world clinical practice rather than a design limitation.

Exclusion criteria included major bleeding within the previous three months, severe heart failure (New York Heart Association class III–IV), severe coronary artery disease (including myocardial infarction or coronary artery bypass grafting within the previous three months), moderate or severe valvular disease, and any condition that contraindicated minimally invasive cardiac surgery.

Baseline clinical data were collected, including demographic characteristics (age, sex, body mass index), cardiovascular risk factors (hypertension, diabetes, smoking history, alcohol use), and comorbidities (coronary artery disease, prior cerebral infarction, hypertrophic cardiomyopathy [HCM], obstructive sleep apnea-hypopnea syndrome [OSAHS]). Laboratory parameters included liver function tests and serum creatinine.

Detailed AF-related history was documented, including AF duration, number of prior CA procedures, and CHA₂DS₂-VASc score.

Echocardiographic parameters obtained from transthoracic echocardiography included: LA anteroposterior diameter (mm), LA transverse and longitudinal diameters (mm), left ventricular (LV) end-diastolic diameter (mm), LV end-systolic diameter (mm), and LV ejection fraction (LVEF, %).

### Ethical approval

The study was approved by the Medical Ethics Committee of Hebei Yanda Hospital (approval number: N-F-20150304). All patients provided informed consent prior to the procedure.

## One-stop hybrid procedure (unilateral thoracoscopic epicardial ablation with percutaneous endocardial ablation)

### Preoperative preparation

Within 2 days prior to the procedure, all patients underwent transthoracic and transesophageal echocardiography to exclude intracardiac thrombi and significant valvular disease. Coronary angiography and pulmonary function tests were performed as indicated. Oral anticoagulants were discontinued 3 days before the procedure and replaced with low-molecular-weight heparin bridging therapy.

### Surgical epicardial ablation

After general anesthesia with double-lumen endotracheal intubation, patients were placed in the right lateral decubitus position. Three left-sided ports were created along the mid-axillary line. The pericardium was opened and suspended, and the oblique sinus was developed to expose the left and right pulmonary vein (PV) antra. The right phrenic nerve was identified and preserved throughout.

A bipolar radiofrequency clamp (AtriCure) was used to create circumferential lesions around each ipsilateral PV pair (12 applications per pair). Conduction block was confirmed by demonstrating exit or entrance block. A linear “roof line” lesion was created connecting the superior PVs using the bipolar clamp. A monopolar ablation pen (AtriCure) was used to create additional posterior LA lesions, ablate the ligament of Marshall and epicardial autonomic ganglia, and create a linear lesion from the left superior PV to the left atrial appendage (LAA) base.

The LAA was excised using an Endo GIA stapler (Covidien) or closed with a clip (AtriClip) based on anatomical suitability. A chest tube was placed, and the chest was closed. Immediately after creation, bidirectional block across this linear lesion was confirmed by differential pacing from both sides of the line.

### Percutaneous endocardial mapping and ablation

Following epicardial ablation, biatrial electroanatomical mapping was performed using the CARTO 3 system (Biosense Webster) with a PentaRay catheter. After completion of all epicardial and endocardial lesions, high-density mapping was repeated to reconfirm bidirectional block across all linear lesions. PV isolation and the integrity of posterior wall lesions were verified. Conduction gaps were ablated using an open-irrigated tip catheter (SmartTouch, 30–35 W, 43 °C). Linear ablation was performed to achieve bidirectional block across the cavotricuspid isthmus and, when indicated, the mitral isthmus.Mitral isthmus line ablation was performed in 7/44 patients (15.9%) for the following indications: perimitral flutter (intraprocedural or preoperative), persistent AF after box isolation, or low-voltage zones at the mitral annulus. Bidirectional block was confirmed by pacing in all 7 patients.

During ongoing AF, high-density maps were created to identify areas with continuous low-voltage signals (< 0.3 mV), rapid local deflections, or spatio-temporal dispersion [9, 10]. Ablation targeting these areas was performed with the endpoint of acute AF termination. Mapping and ablation of organized atrial tachycardia (AT) were performed when present.

The hybrid procedure was performed by a dedicated heart team consisting of cardiac surgeons and electrophysiologists working in the same operative suite.

### Restoration of sinus rhythm

Patients remaining in AF received ibutilide and underwent electrical cardioversion (ECD). Bidirectional block was confirmed by differential pacing. Antiarrhythmic drugs (AADs) were continued for 3 months post-operatively.

### Periprocedural and long-term anticoagulation management

Warfarin or DOACs were stopped 48 h pre-procedure, with bridging if needed. Intraprocedural heparin maintained ACT > 300 s. Anticoagulation resumed 24–48 h post-procedure after hemostasis. Long-term OAC was guided by CHA₂DS₂-VASc score and shared decision-making, irrespective of rhythm. Discontinuation after LAA exclusion was individualized.

### Follow-up

Follow-up was conducted by dedicated research personnel via telephone/WeChat at 1, 3, 6, 12 months and annually thereafter. Patients were asked to report any symptoms or documented arrhythmias from local hospitals. Whenever possible, 12-lead ECG or 24-hour Holter was performed during in-hospital visits. Original tracings were not centrally archived for out-of-town patients, but recurrence events were confirmed by clinical reports or ECG documentation from local hospitals. No patient received an implantable loop recorder or CIED. AADs, primarily amiodarone, were prescribed for all patients during the 3-month blanking period. After the blanking period, AADs discontinuation was encouraged unless clinically indicated for recurrent arrhythmias. Recurrence status was reported as freedom from AF/ Atrial Flutter (AFL)/AT after the blanking period, irrespective of AAD use. All 44 patients completed the median 5.2-year follow-up with no loss to follow-up.

As rhythm surveillance relied on intermittent outpatient ECG/Holter instead of continuous long-term monitoring, asymptomatic silent arrhythmia episodes may be missed, potentially underestimating true arrhythmia recurrence burden.

### Statistical analysis

Given the exploratory design and limited sample size (44 patients, 22 events), Cox proportional hazards regression was used to identify factors associated with AF recurrence. To minimize the risk of model overfitting given the limited number of events, the multivariable model included only four prespecified covariates: preoperative LA diameter, diabetes mellitus, AF duration, and number of prior endocardial ablations. Results are presented as hazard ratios (HRs) with 95% confidence intervals (CIs) and should be interpreted as hypothesis-generating rather than confirmatory. The proportional hazards assumption was tested using Schoenfeld residuals. Recurrence-free survival was estimated using the Kaplan–Meier method, and differences between groups were compared using the log-rank test.

All statistical tests were two-tailed, and a P value < 0.05 was considered statistically significant. Statistical analyses were performed using R software (version 4.3.2) .

## Results

### Baseline characteristics

A total of 44 patients with PsAF or LS-PsAF were enrolled. The cohort was predominantly male (77.3%), with a mean age of 59.6 ± 8.3 years. The median AF duration was 8.0 years (IQR: 4.0–13.0). Notably, 97.7% of patients had undergone prior CA (mean 1.4 ± 0.7 procedures). The mean LA anteroposterior diameter was 45.0 ± 6.4 mm, and the mean CHA₂DS₂-VASc score was 2.2 ± 1.5. Detailed baseline characteristics are presented in Table 1.

**Table 1:** Baseline Characteristics of the Study Population

Characteristic	Total (*N* = 44)
**Demographic and Clinical History**	
Male, n (%)	34 (77.3)
Age (years), mean ± SD	59.6 ± 8.3
Hypertension, n (%)	20 (45.5)
Diabetes, n (%)	13 (29.5)
Cerebral infarction, n (%)	6 (13.6)
Coronary artery disease, n (%)	19 (43.2)
Renal dysfunction, n (%)	4 (9.1)
Hypertrophic cardiomyopathy, n (%)	6 (13.6)
OSAHS, n (%)	3 (6.8)
Smoking history, n (%)	16 (36.4)
Drinking history, n (%)	13 (29.5)
BMI (kg/m²), mean ± SD	26.7 ± 3.5
LDL-C (mmol/L), mean ± SD	2.56 ± 0.6
ALT (U/L), median (IQR)	24.00 (24.73)
Creatinine (µmol/L), median (IQR)	75.25 (91.25)
**Cardiac and Echocardiographic Parameters**	
Duration of atrial fibrillation (years), median (IQR)	8.0 (4.0–13.0)
Prior catheter ablation, n (%)	43 (97.7)
Number of previous catheter ablations, mean ± SD	1.4 ± 0.7
CHA₂DS₂-VASc score, mean ± SD	2.2 ± 1.5
LA diameter—anteroposterior (mm), mean ± SD	45.0 ± 6.4
LA diameter—left–right (mm), mean ± SD	45.5 ± 4.8
LAdiameter—superior–inferior (mm), mean ± SD	60.9 ± 6.6
LV end-diastolic diameter (mm), mean ± SD	48.2 ± 4.1
LV end-systolic diameter (mm), mean ± SD	31.1 ± 5.6
LVEF (%), mean ± SD	62.6 ± 9.3

Values are presented as n (%), mean ± standard deviation, or median (interquartile range). OSAHS: obstructive sleep apnea-hypopnea syndrome; BMI: body mass index; LDL-C: low-density lipoprotein cholesterol; ALT: alanine aminotransferase; SD: standard deviation; IQR: interquartile range; LA: left atrium; LV: left ventricular; LVEF: left ventricular ejection fraction

### In-hospital outcomes

**Fig. 1:**
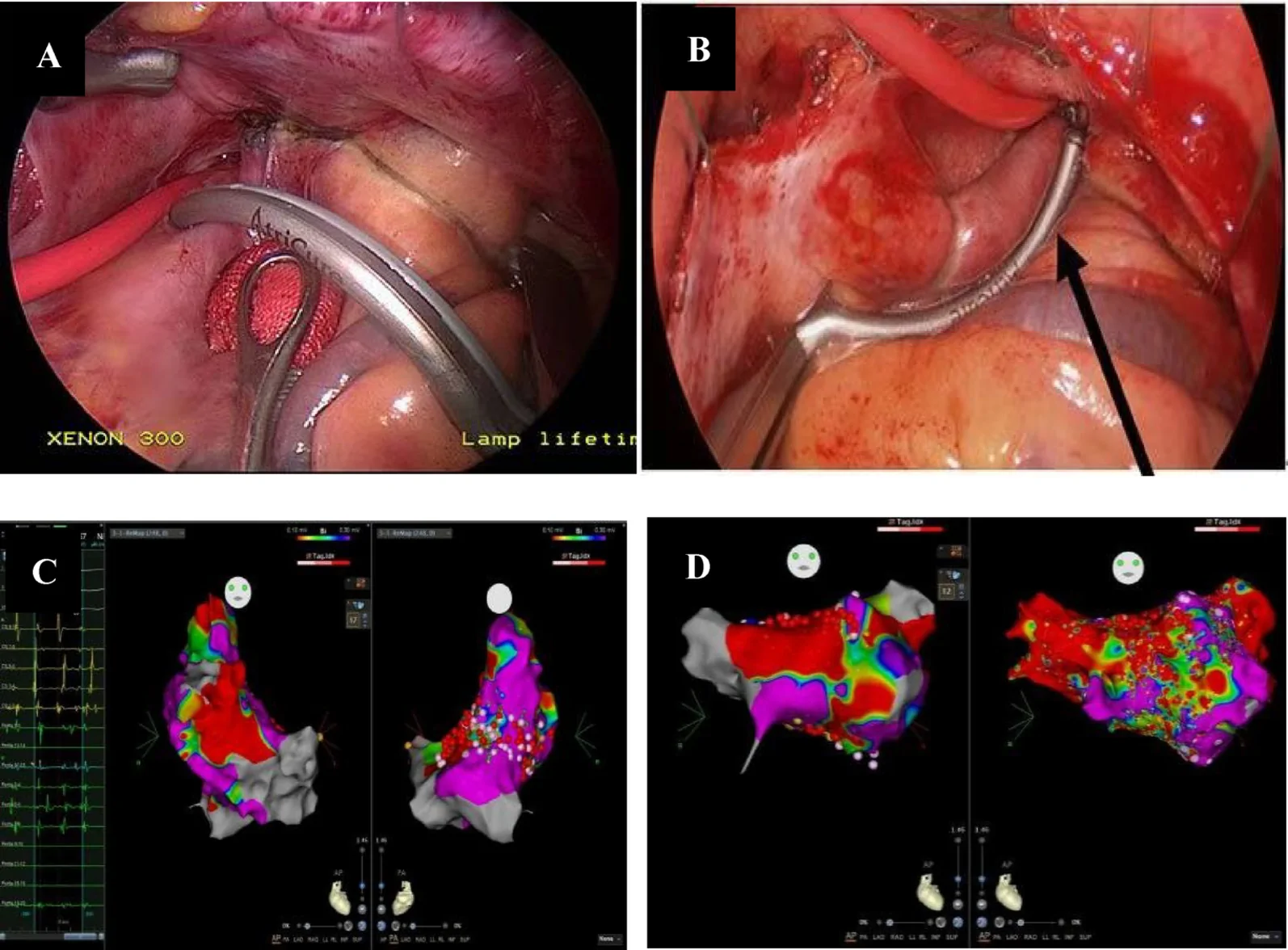
Unilateral Thoracoscopic Epicardial Ablation / Percutaneous Endocardial Ablation. (**A**) Left pulmonary vein (PV) isolation was performed with 12 applications per vein using a bipolar radiofrequency clamp (AtriCure, USA). (**B**) Right PV isolation was similarly performed with 12 applications per vein using the same clamp. (**C**) Catheter ablation sites in one patient included the right atrial septum and posterior wall, extending to the middle of the border ridge (views: AP and PA). (**D**) Ablation at the top of the left atrium (LA) connected the right pulmonary artery orifice to the left atrial appendage orifice; additional ablation was performed at the bottom of the LA (views: AP and AP)

Following surgical epicardial ablation, only 2 patients converted to sinus rhythm (SR); 36 remained in AF, 5 in AFL, and 1 had alternating AF/AFL. Subsequent endocardial mapping and ablation restored SR in an additional 31 patients, and 11 converted after antiarrhythmic drugs or ECD. Bidirectional block was confirmed in all patients achieving SR. During the perioperative period, 27 patients (61.4%) maintained SR throughout hospitalization. By discharge, after treatment with amiodarone or cardioversion, 41 patients (93.2%) had achieved and maintained SR. Two patients (4.5%) remained in persistent AF, and 1 (2.3%) in AFL (Fig. 1).

**Fig. 2:**
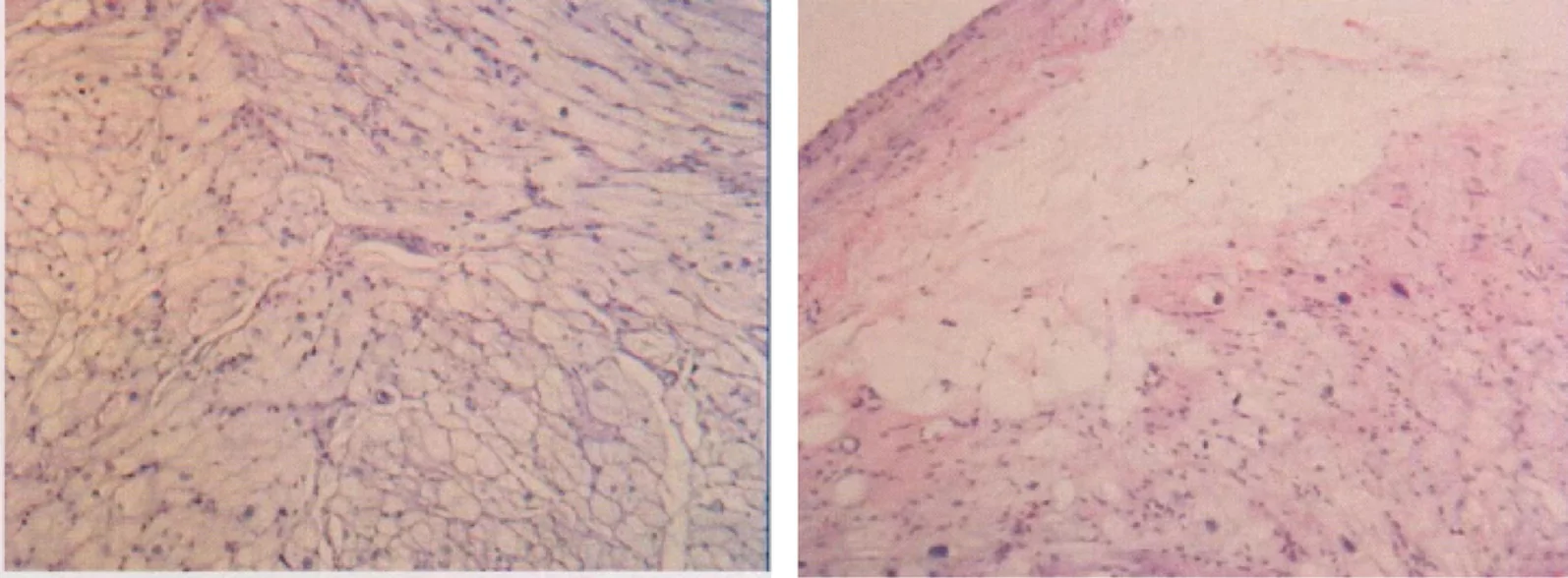
**A**, **B**. Histopathological features of the left atrial appendage. Sections demonstrate diffuse cardiomyocyte hypertrophy, focal endocardial thickening, vacuolar degeneration of cardiomyocytes, mild interstitial inflammatory cell infiltration, and subendocardial adipose tissue hyperplasia

The LAA was resected in 41 patients and clipped in 3. Pathological examination of LAA specimens was performed in 40 patients (three patients underwent LAA clipping without tissue resection, and one patient had a missing pathological report). Among these 40 specimens, cardiomyocyte hypertrophy was observed in 37 patients (92.5%), endocardial thickening in 30 patients (75.0%), inflammatory cell infiltration in 29 patients (72.5%), and myocardial fibrosis in 6 patients (15.0%). Notably, occult thrombus was identified in 4 patients (10.0%) despite negative preoperative transesophageal echocardiography, suggesting preliminary histological rationale for LAA intervention, and further controlled research is needed to clarify the clinical value of routine LAA management. (see Supplementary Tables 2 and Fig. 2). Light microscopy of the LAA specimens showed mural thrombi in various stages of organization in all cases. No acute fresh thrombi were identified; instead, the thrombi exhibited features of subacute to chronic evolution, including partial red blood cell lysis, ingrowth of fibrous tissue from the endocardium, and associated endocardial thickening and interstitial lymphocytic infiltration.

### Three-month blanking period outcomes

**Fig. 3:**
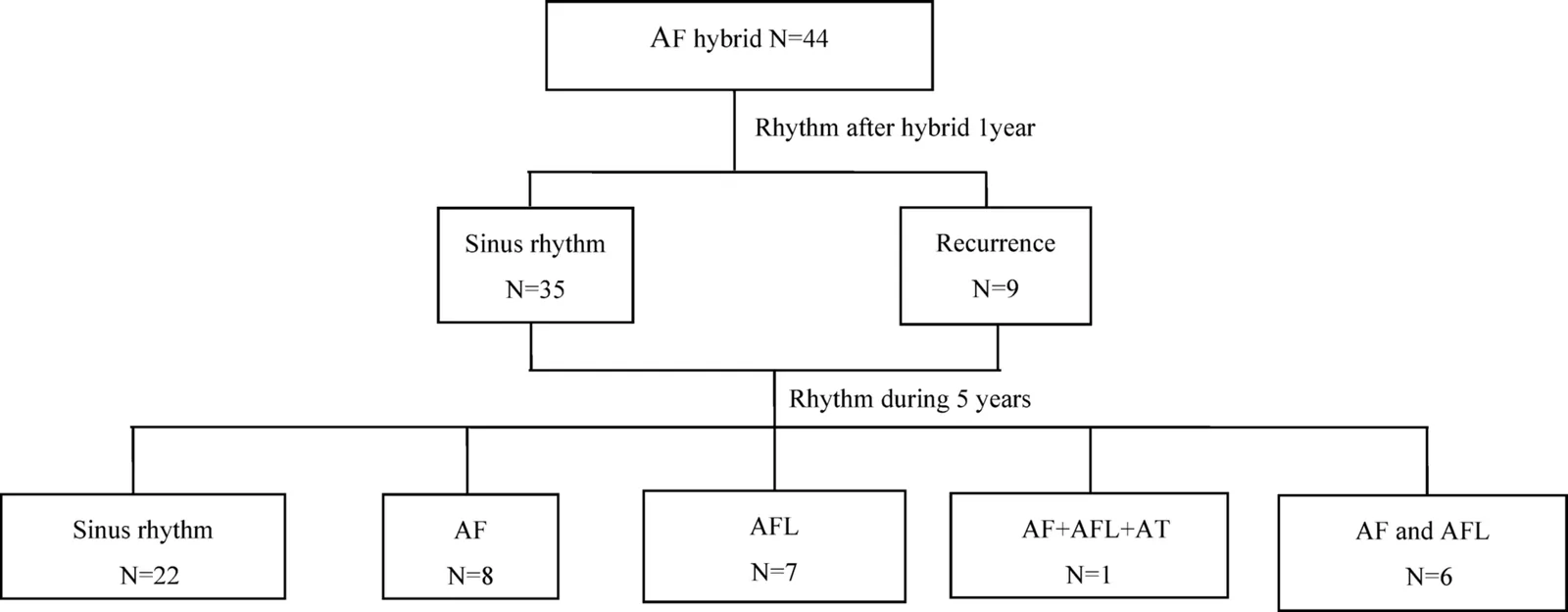
Flowchart of Cardiac Rhythm Changes Following the Hybrid Procedure. AF: atrial fibrillation; AFL: atrial flutter ; AT: atrial tachycardia; ECD: Electric cardioversion

During the 3-month blanking period, 35 patients (79.5%) remained SR free from AADs. Among the 9 patients with early recurrence, 2 exhibited persistent AF, 3 paroxysmal AF, 2 paroxysmal AFL, and 2 both AF and AFL (Fig. 3).

### Long-term efficacy and recurrence patterns

**Fig. 4:**
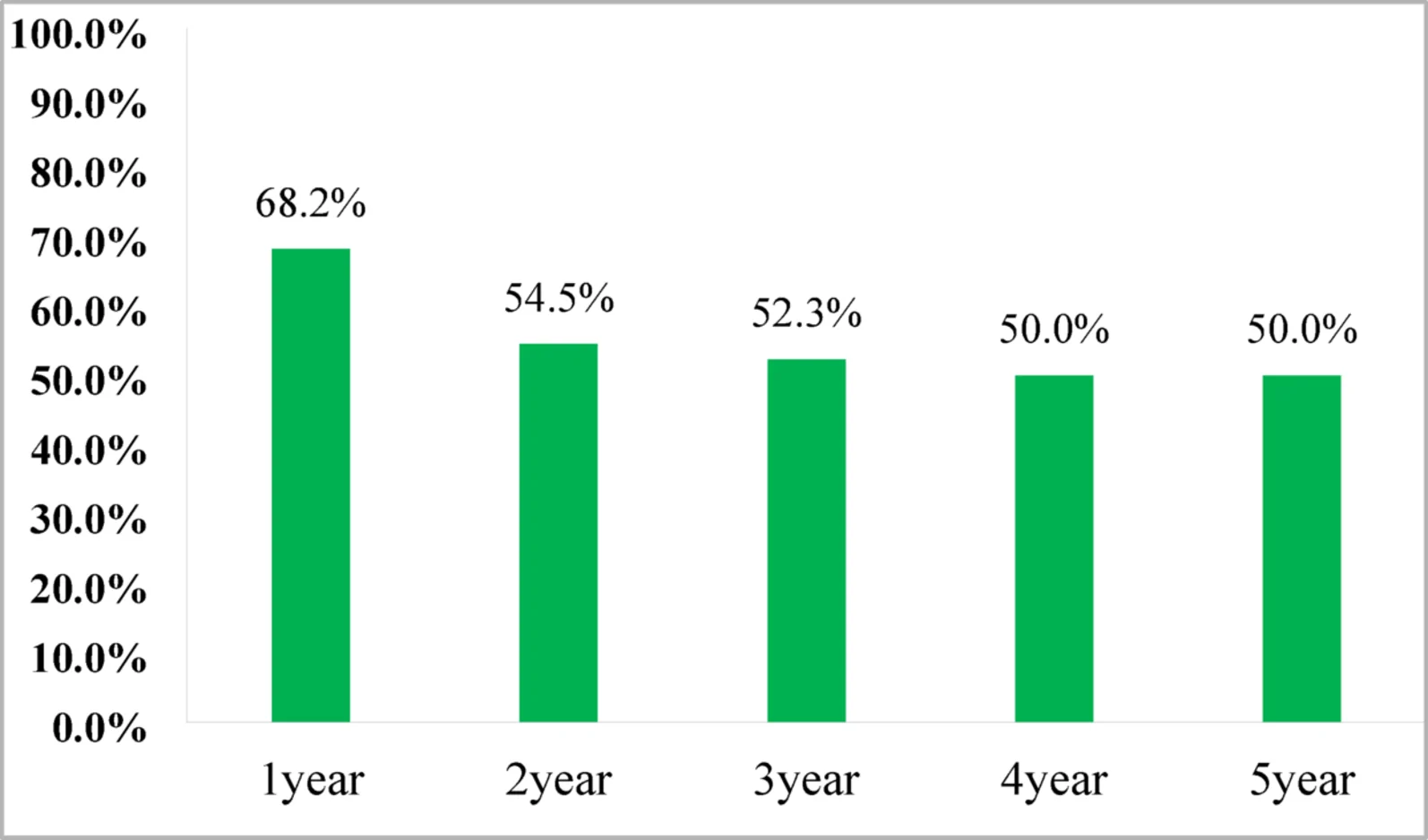
Freedom from Atrial Fibrillation Over Time Following the Hybrid Procedure. The freedom from atrial arrhythmias was 68.2% at 1 year, 54.5% at 2 years, 52.3% at 3 years, 50.0% at 4 years, and 50.0% at 5 years

Over a median follow-up of 5.2 years, sinus rhythm was maintained in 22 patients (50.0%) based on the primary endpoint definition (regardless of AAD use). Of these 22 patients, 21 (95.5%) remained free from any atrial arrhythmia without antiarrhythmic drugs (off AAD), accounting for 47.7% (21/44) of the entire cohort. Only 1 patient (4.5% of the success group) continued AADs without documented recurrence (on AAD). The freedom from AF/AFL/AT at 1, 2, 3, 4, and 5 years was 68.2%, 54.5%, 52.3%, 50.0%, and 50.0%, respectively (Fig. 4). Recurrence occurred in 22 patients, with AF being the most common presenting arrhythmia (18.2%). Analysis of recurrence timing revealed that 63.6% of recurrences (14/22) occurred within the first postoperative year, followed by 27.3% (6/22) in the second year, and 4.5% (1/22) in each of the third and fourth years (see Fig. 3 and Supplementary Table 4).

### Antithrombotic therapy during follow-up in patients with recurrence

Among the 22 patients with documented AF recurrence (median CHA₂DS₂-VASc score 2, IQR 2–3), all patients were prescribed oral anticoagulation at hospital discharge, while antithrombotic status was re-evaluated at a median follow-up of 5.2 years. Antithrombotic status at final follow-up was ascertained via patient or family report during telephone or WeChat interviews. Among the 22 patients with AF recurrence, three had died by final follow-up. Of the surviving 19 patients, only 3 (15.8% of survivors, 13.6% of the total recurrence cohort) remained on long-term oral anticoagulation at last contact. Antithrombotic regimens could not be definitively confirmed for the remaining 16 survivors (72.7% of all recurrent patients): some potentially received single antiplatelet therapy, whereas others received no long-term antithrombotic medication. Despite low rates of persistent guideline-directed OAC, no thromboembolic complications occurred throughout follow-up.

### Predictors of AF recurrence

In this hypothesis-generating exploratory multivariate Cox regression analysis (limited by only 22 events for four covariates), preoperative LA anteroposterior diameter showed a nominal borderline association with AF recurrence (HR = 1.073, 95% CI: 1.001–1.151, *P* = 0.047). Although the confidence interval is relatively narrow, the lower bound is very close to 1.0 (1.001) and the P-value is marginal (0.047). Given the limited sample size (44 patients, 22 events) and the exploratory nature of the analysis, this finding should be interpreted with caution and requires validation in larger cohorts, Table 2.

**Table 2:** Multivariate Cox Regression Analysis for Predictors of Atrial Fibrillation Recurrence

Variable	HR	95% CI	*P*-value
Preoperative LA anteroposterior diameter	1.073	1.001–1.151	0.047
Diabetes mellitus	2.447	0.860–6.963	0.093
Preoperative AF duration (years)	1.011	0.945–1.082	0.757
Number of prior endocardial ablations	0.995	0.500–1.981	0.989

Proportional hazards assumption (PH test): All *P* > 0.05 (global *P* = 0.89). Exploratory analysis with limited events (*n* = 22 for 4 covariates). Although the confidence interval for LA diameter is relatively narrow (1.001–1.151), the lower bound approaches 1.0 and the P-value is marginal (0.047); these findings should be interpreted as hypothesis-generating and require validation in larger cohorts. LA: left atrium; AF: atrial fibrillation

The proportional hazards assumption was satisfied for all variables (all *P* > 0.05), confirming the validity of the model. However, given the exploratory nature of the analysis and the limited event-to-covariate ratio (22 events for 4 covariates), these results should not be overinterpreted as definitive evidence of independent prediction.

### Management of recurrence and long-term prognosis

Among the 22 patients with recurrence, 12 underwent repeat CA, two-thirds of these patients (66.6%) successfully restored SR, with pulmonary veins remaining the predominant target (Supplementary Table 5). Some patients received electrical or pharmacological cardioversion or antiarrhythmic medications.

**Fig. 5:**
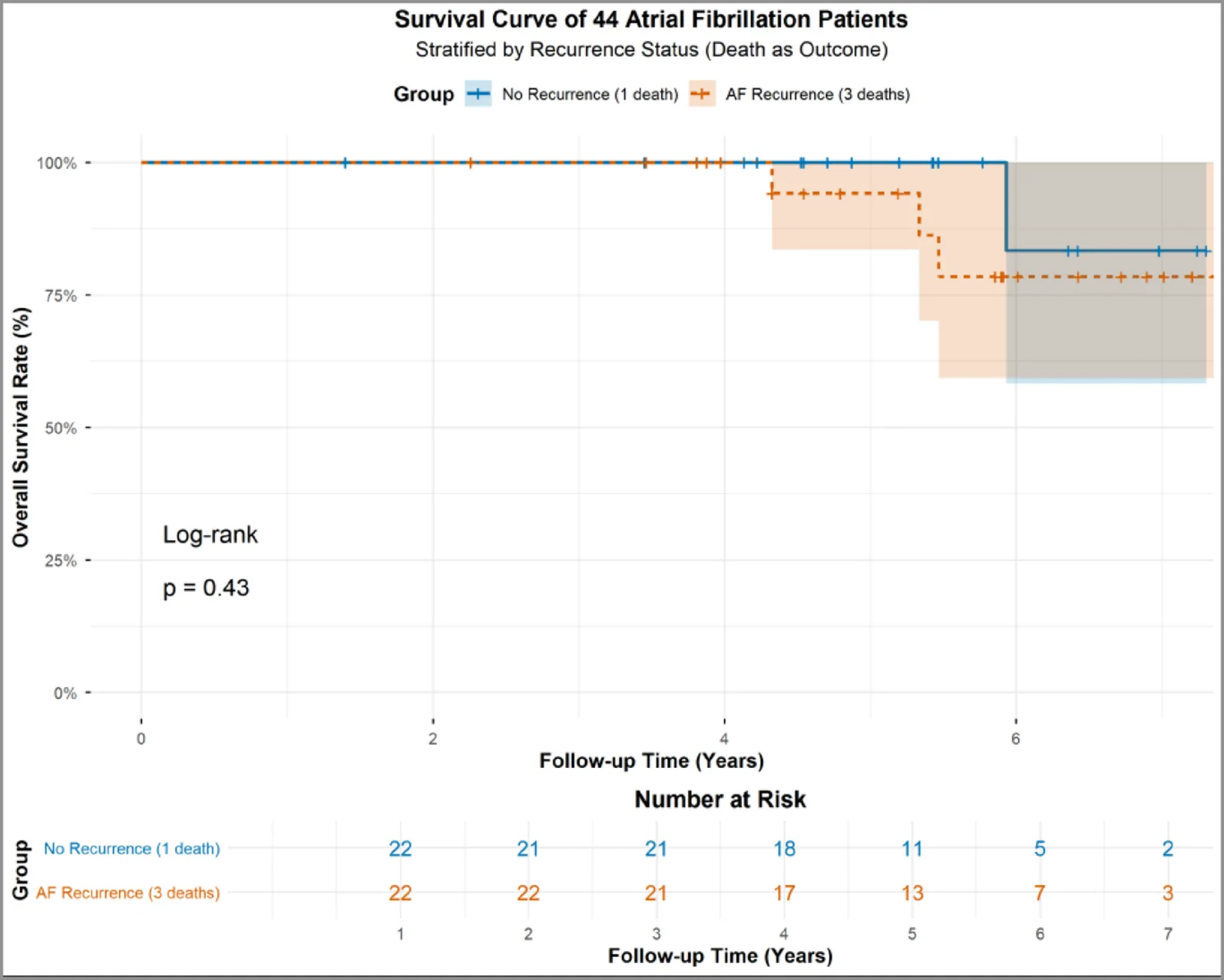
Kaplan–Meier Survival Estimates for All-Cause Mortality Stratified by AF Recurrence Status. Kaplan–Meier curves showing all-cause mortality in patients with and without atrial fibrillation (AF) recurrence during follow-up. There was no significant difference in all-cause mortality between the two groups (log-rank *P* = 0.43); AF: atrial fibrillation

Over a median follow-up period of 5.2 years, 4 patients (9.1%) died, including one death attributable to cardiac causes and one due to sepsis. The all-cause mortality rate was 4.5% in the No Recurrence group (1/22) and 13.6% in the AF Recurrence group (3/22). Heart failure (HF) occurred in 2 patients, and 4 patients required pacemaker implantation (Supplementary Table 6). Kaplan-Meier analysis revealed no statistically significant association between all-cause mortality and AF recurrence (log-rank *P* = 0.43). Given the small number of deaths (*n* = 4), this analysis was exploratory and underpowered for definitive conclusions, Fig. 5. Notably, no thromboembolic events were observed during follow-up.

Besides, we analyzed the association between acute termination of AF during procedure and post-procedural AF recurrence. A total of 44 patients were divided into two groups: 33 patients who achieved sinus rhythm immediately after ablation and 11 patients who required pharmacological or electrical cardioversion. The chi-square test showed no significant difference in the overall recurrence rate between the two groups (χ2 = 0.485, *P* = 0.486). Log-rank test revealed no significant difference in recurrence-free survival between groups (χ2 = 0.60, *P* = 0.500). Univariate Cox regression analysis demonstrated that failure to acutely terminate AF was not associated with increased risk of AF recurrence (HR = 1.408, 95%CI: 0.566–3.505, *P* = 0.462).

## Discussion

This retrospective study provides long-term (median 5.2 years) outcome data on a one-stop unilateral thoracoscopic hybrid ablation approach in a challenging cohort of PsAF or LS-PsAF and prior failed CA. The unilateral left-sided thoracoscopic approach offers a less invasive alternative to bilateral access while still enabling comprehensive posterior LA box isolation, ligament of Marshall ablation, and LAA management [3, 4].First, the procedure demonstrated an acceptable safety profile with no in-hospital mortality and a low rate of major complications (6.8% pericardial effusion). Second, despite the complex patient population, SR was maintained in half of the patients at 5 years (50.0%), with most recurrences (63.6%) occurring within the first postoperative year. Third, LA anteroposterior diameter showed a potential association with recurrence in exploratory analysis, suggesting its potential predictive value for recurrence, although this finding is limited by the small sample size. Fourth, no thromboembolic events were observed during the median 5.2-year follow-up, providing preliminary evidence for the efficacy of LAA intervention; however, these results need further validation in large controlled studies due to the small sample size and lack of systematic rhythm monitoring.

The 5-year freedom from atrial arrhythmia rate of 50.0% in our study is consistent with the reported range for hybrid ablation of persistent AF [13–15].Direct comparison with a control group is lacking in this single-arm study. However, published outcomes of catheter ablation in persistent AF populations provide useful benchmarks. Luo et al. reported 5-year freedom from atrial tachyarrhythmias of 40.7% to 47.3% after a single ablation procedure in patients with persistent AF [16]. Our 1-year freedom from AF/AFL/AT rate of 68.2% is comparable to the 67.7% primary effectiveness rate reported in the CONVERGE trial at 12 months [17]. Li et al., in a multicenter cohort study of 887 patients with persistent AF, demonstrated 12-month freedom from AF/AT of 68.9% in patients with intraprocedural AF termination and 58.2% in those without [18]. The relatively high recurrence rate in our cohort can be explained by the high-risk characteristics of enrolled patients, including long-standing AF (median 8.0 years), enlarged LA (45.0 ± 6.4 mm), and a nearly universal history of failed prior CA (97.7%), all of which are validated risk factors for AF recurrence [19–21]. Our exploratory multivariate Cox regression found a borderline correlation between enlarged LA diameter and AF recurrence; however, due to limited event count, this finding is not sufficient to confirm LA dimension as an independent prognostic predictor for recurrence and requires external validation.

The mechanisms underlying recurrence in this population are likely multifactorial. First, extrapulmonary vein triggers and substrate play a critical role. While PVI alone is effective for paroxysmal AF, the initiation and perpetuation of PsAF and LS-PsAF increasingly involve non-PV triggers and an abnormal atrial substrate. In our study, the left-sided unilateral thoracoscopic epicardial ablation, while effective for creating a posterior LA box lesion and targeting the ligament of Marshall and epicardial autonomic ganglia, did not address other known arrhythmogenic structures such as the superior vena cava, coronary sinus, crista terminalis, or Bachmann’s bundle.

Second, the atrial substrate itself is a key determinant. The identification of LA diameter as a potential predictor (in our exploratory analysis) aligns with large meta-analyses demonstrating that LA enlargement consistently predicts poorer outcomes after ablation [19–21]. Pathological examination of resected LAA specimens provided indirect evidence of this substrate, revealing myocardial hypertrophy, endocardial thickening, and inflammatory cell infiltration in a majority of patients. Notably, myocardial fibrosis was present in 13.6% of patients, and five of these six patients experienced AF recurrence. This indicates that advanced atrial structural remodeling and fibrosis, which are poorly targeted by current ablation techniques, are key targets for future procedural optimization [22–24].

Although only 2/44 patients (4.5%) converted after epicardial ablation alone, the epicardial phase in our protocol accomplished PVI, roof line, posterior wall lesions, Marshall ligament ablation, autonomic ganglion ablation, LSPV-to-LAA linear lesion, and LAA management—components difficult to achieve endocardially. These interventions modified the atrial substrate and facilitated subsequent endocardial mapping and gap closure. Therefore, the low acute conversion rate does not negate the value of epicardial ablation. The epicardial and endocardial ablation phases exert synergistic effects, jointly contributing to the 50% 5-year rhythm control success rate.

Third, procedural timing warrants consideration. Our one-stop, same-day approach contrasts with staged hybrid procedures. Richardson and colleagues [25]reported that staged approaches increase detection of incomplete PVI during subsequent endocardial mapping, suggesting that acute inflammation from the surgical stage may temporarily mask gaps—though this did not prolong time to first recurrence. While our same-day strategy offers the advantage of addressing both epicardial and endocardial components in a single session, it does not eliminate PV reconnection [26, 27]. Among 12 patients who underwent repeat CA for recurrence, two-thirds (66.6%) successfully restored SR, with pulmonary veins remaining the predominant target—suggesting that repeat intervention can be beneficial.

Fourth, occult LAA thrombus was detected in 10.0% (4/40) of patients with negative preoperative TEE results. This highlights an inherent limitation of TEE in clinical practice, as small and chronic thrombi hidden among LAA trabeculations are frequently missed, particularly in patients with persistent AF. Histopathological features characterized by fibrous ingrowth and endocardial reaction further confirmed the chronic or subacute nature of these thrombi, excluding a procedural origin. Although no subsequent thromboembolic events occurred in these patients, this preliminary finding based on a small case cohort (*n* = 4) needs further verification in larger-scale studies. Collectively, these histological findings support the potential value of prophylactic LAA intervention.The low long-term oral anticoagulation persistence rate observed in recurrent patients, alongside zero observed thromboembolic events during follow-up, provides preliminary clinical hints that routine intraoperative LAA exclusion may mitigate stroke risk and partially offset the risks related to suboptimal outpatient anticoagulation adherence.Large prospective controlled trials are warranted to confirm whether routine LAA exclusion provides additional antithrombotic benefits over standard guideline-based anticoagulation.

In conclusion, recurrence after hybrid ablation for persistent AF is likely a consequence of residual non-PV triggers, an advanced and incompletely modified atrial substrate (including fibrosis), and possibly late PV reconnection. The absence of thromboembolic events in our cohort is encouraging, but the 50.0% recurrence rate in this challenging group underscores that achieving long-term SR remains a significant challenge. While our findings are hypothesis-generating, they suggest that future studies should focus on more comprehensive substrate modification and optimized patient selection to improve outcomes in this challenging population.

### Limitations

This study has several limitations. First, as a retrospective single-center study, it is subject to selection bias and lacks a control group. However, the cohort represents a real-world Asian population with complex persistent AF and prior failed ablation—a group often underrepresented in clinical trials. Given the logistical demands of coordinating cardiac surgery, electrophysiology, and anesthesiology teams for a same-day hybrid procedure—resources not universally available—this single-center experience provides valuable insights despite its moderate sample size. Second, the unilateral thoracoscopic approach does not constitute a full bi-atrial Cox-maze IV lesion set. Third, the limited sample size (*n* = 44, with 22 events) constrained statistical power; therefore, the multivariable Cox model was exploratory and the results should not be interpreted as definitive evidence of independent prediction.Additionally, the low event-to-covariate ratio in the Cox regression model may lead to model instability and overfitting, further limiting the reliability of the borderline correlation between LA diameter and AF recurrence. Fourth, recurrence was determined from clinical reports and ECGs rather than continuous monitoring, which may have underestimated the true recurrence burden. Fifth, the absence of thromboembolic events, while encouraging, requires confirmation in larger studies.

### Conclusion

In this exploratory analysis of selected patients with PsAF/LS-PsAF and prior failed CA, one-stop hybrid ablation with routine LAA management appeared safe and was associated with rhythm control in half of patients at 5 years. Exploratory analysis suggested a borderline correlative trend between larger preoperative LA diameter and arrhythmia recurrence; this preliminary finding requires external validation and cannot confirm the independent predictive value of LA diameter at present.These long-term data suggest hybrid ablation is a viable strategy, but larger prospective studies are required for confirmation.

## Supplementary Information

Supplementary Material 1

## Data Availability

No datasets were generated or analysed during the current study.
