# Supplementary material for: Long-term outcomes of one-stop unilateral thoracoscopic hybrid ablation for persistent atrial fibrillation after failed catheter ablation
Source: J Cardiothorac Surg. 2026 Jun 25;21:687. doi: 10.1186/s13019-026-04441-x (PMC13560335; doi:10.1186/s13019-026-04441-x)
Supplement: Supplementary file 1 [file 13019_2026_4441_MOESM1_ESM.docx]

**Supplemental material**

**Supplement table 1. Price of operation**

| **Price of operation** | **Result** |
| --- | --- |
| Hospitalization days | 19.2±7.2 |
| Average hospitalization expenses (RMB),media | 187990 |
| Anesthesia duration (min) | 429.5±83.6 |
| Operation duration (min) | 309.1±62.9 |
| Operation blood loss (ml),media | 100 |
| Endotracheal intubation (day),media | 1.0 |
| Drainage days (day),media | 4.0 |
| Quantity of drainage(ml),media | 780 |

**Supplement table 2. Pathology of left atrial appendage**

| **Pathology of left atrial appendage^＊^** | **Result(N=40)** |
| --- | --- |
| Cardiomyocyte hypertrophy | 37(92.5) |
| Vacuolar degeneration of myocardial cells | 11(27.5) |
| Cellular fat and mucus degeneration | 6(15.0) |
| Other degeneration | 6(15.0) |
| Myocardial fibrosis | 6(15.0) |
| Endocardial thickening | 30(75.0) |
| Inflammatory cell infiltration | 29(72.5) |
| Thrombus | 4( 10.0) |
| Other pathological manifestations | 23( 57.5) |

**^＊^**Three patient**^＊^**Three patients underwent surgical clipping of the left atrial appendage, and one patient had a pathological report missing.

**Supplement table 3. Catheter ablation site**

| **Catheter ablation site** | **Result(N=43)** |
| --- | --- |
| Left atrium(n,%) |  |
| Right pulmonary vein | 35( 81.4) |
| Left pulmonary vein | 4(9.3 ) |
| Mitral annulus | 6(14.0) |
| Left atrial appendage | 4( 9.3) |
| Left atrial others | 26(60.5) |
| Right atrium(n,%) |  |
| Tricuspid annulus/isthmus | 23(57.5) |
| Superior vena cava | 3(7.0) |
| Inferior vena cava | 3(7.0 ) |
| Auricula dextra | 3(7.0) |
| Right atrial others | 13(30.2) |
| Ostium of coronary sinus(n,%) | 2(4.7) |

**Supplement table 4. Recurrence time after hybrid**

| **Recurrence time^＊^** | **Result**  **(N=22)** | **Freedom fromAF**  **(N=44)** |
| --- | --- | --- |
| Recurrence within 1 year (n,%) | 14(63.6) | 30(68.2) |
| Recurrence within 2 year (n,%) | 6(27.3) | 24(54.5) |
| Recurrence within 3 year (n,%) | 1(4.5) | 23(52.3) |
| Recurrence within 4 year (n,%) | 1(4.5) | 22(50.0) |
| Recurrence within 5 year (n,%) | 0(0) | 22(50.0) |

**^＊^**The average recurrence duration is (5, 20.5) months.

**Supplement table 5. Re-catheter ablation site**

| **Re-catheter ablation site^＊^** | **Result(N=10)** |
| --- | --- |
| Left atrium (n,%) |  |
| Pulmonary vein | 8(80) |
| Mitral isthmus | 4( 40) |
| Left atrial appendage | 4(40 ) |
| Left atrial septum | 3( 30) |
| Left atrial others | 6(60 ) |
| Right atrium (n,%) |  |
| Isthmus of tricuspid valve | 3(30 ) |
| Superior and inferior vena cava | 1(10) |
| Auricula dextra | 2(20 ) |
| Right atrial others | 4( 40) |
| Coronary sinus (n,%) | 1(10) |

**^＊^**A total of 12 patients underwent radiofrequency ablation again, of which 2 patients missed the radiofrequency data.

**Supplement table 6. Major Adverse Cardiovascular Events**

| **Major Adverse Cardiovascular Events^＊^** | **Result(N=44)** |
| --- | --- |
| Recurrent cerebral infarction (n,%) | 0(0) |
| Acute myocardial infarction (n,%) | 0(0) |
| Heart failure (n,%) | 2(4.5) |
| Death (n,%) | 4(9.1) |
| Haemorrhage (n,%) | 0(0) |
| Implantation of pacemakers (n,%) | 4(9.1) |

**^＊^**The median follow-up time was 62.5 months, equivalent to 5.2 years.

Supplement **Figure 1.** Rhythm within 3 months after surgery

Rhythm within 3 months after hybrid

|  |  |
| --- | --- |
| Sinus rhythm | 34 |
| Persistent AF | 2 |
| Paroxysmal AF | 3 |
| Paroxysmal atrial flutter | 2 |
| Paroxysmal atrial tachycardia | 1 |
| Paroxysmal AF and atrial flutter | 2 |

AF:atrial fibrillation

**Supplement Figure 2.** Postoperation rhythm after 3 months

Postoperation rhythm after 3 months

|  |  |
| --- | --- |
| Sinus rhythm | 21 |
| AF | 9 |
| AF and atrial tachycardia | 1 |
| AF and atrial flutter | 8 |
| Atrial flutter | 5 |

AF:atrial fibrillation

**Supplement Figure 3. Relationship between rhythm during hospitalization and after 3 months,P=0.038.**

Relationship between rhythm during hospitalization and after 3 months

|  | Recurrence after 3 months | After 3 months without recurrence |
| --- | --- | --- |
| Sinus rhythm | 12 | 15 |
| Atrial tachycardia | 2 | 1 |
| AF | 4 | 2 |
| Atrial flutter | 4 | 1 |
| AF and atrial flutter | 0 | 1 |

AF:atrial fibrillation
